# Artificial gravity as a potential countermeasure for Spaceflight Associated Neuro-Ocular Syndrome

**DOI:** 10.1038/s41433-024-03178-y

**Published:** 2024-06-14

**Authors:** Ethan Waisberg, Joshua Ong, Mouayad Masalkhi, Kazuhito Shimada, Andrew G. Lee

**Affiliations:** 1 Department of Clinical Neurosciences, University of Cambridge, Cambridge, UK; 2 Michigan Medicine, University of Michigan, Ann Arbor, MI USA; 3 University College Dublin School of Medicine, Belfield, Dublin Ireland; 4 TSUKUBA KOKEN, Tsukuba, Japan; 5 Department of Ophthalmology, Blanton Eye Institute, Houston Methodist Hospital, Houston, TX USA; 6 The Houston Methodist Research Institute, Houston Methodist Hospital, Houston, TX USA; 7 Departments of Ophthalmology, Neurology, and Neurosurgery, Weill Cornell Medicine, New York, NY USA; 8 Department of Ophthalmology, University of Texas Medical Branch, Galveston, TX USA; 9 University of Texas MD Anderson Cancer Center, Houston, TX USA; 10 Texas A&M College of Medicine, Bryan, TX USA; 11 Department of Ophthalmology, The University of Iowa Hospitals and Clinics, Iowa City, IA USA

**Keywords:** Eye diseases, Technology

Long-duration spaceflight (LDSF) poses significant challenges on the human body, including the emergence of unique physiological conditions such as Spaceflight-Associated Neuro-Ocular Syndrome (SANS, ex. VIIP). SANS is a complex collection of ocular changes observed in astronauts during and after long-duration space missions [1]. Initially identified through post-flight examinations, SANS includes symptoms such as globe flattening [2], choroidal folds [3], and optic disc oedema [4]. The proposed mechanisms underpinning SANS pathophysiology are not fully clear yet, but include cephalad brain and orbital fluid shift [5, 6], increased levels of radiation [7], intracranial pressure changes, vascular dysregulation, mitochondrial dysfunction [8] and shifts in cerebrospinal fluid dynamics, which collectively may contribute to the ocular changes documented [9].

The unique microgravity conditions in space are a significant factor contributing to SANS pathophysiology, where microgravity causes head-ward or cephalic bodily fluid distribution that may increase intracranial pressure (ICP), which subsequently potentially affects the eyes [4]. This condition highlights the critical need for understanding the adaptive mechanisms of the human body in space in order to protect astronaut health and inform longer-duration spaceflights. In this manuscript, we explore the potential of artificial gravity as a potential countermeasure for SANS, by examining existing studies on AG simulation on Earth and in space and the mechanisms underlying SANS.

**Fig. 1:**
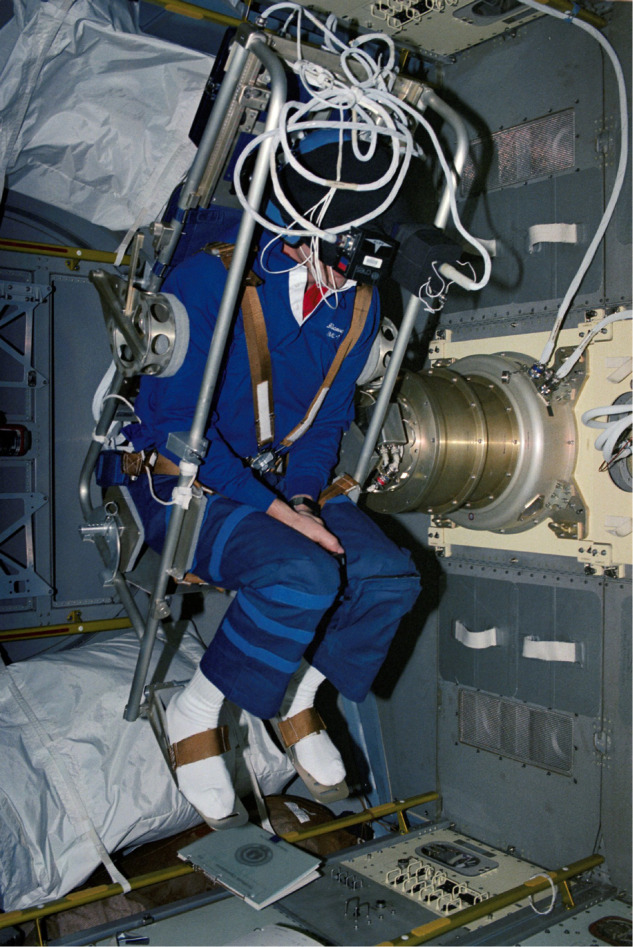
STS-42 IML-1 Space Shuttle Mission Specialist Hilmers in the Microgravity Vestibular Investigation rotating chair. The chair loaded almost 1 G to a subject in various positions but was not intended to be an Artificial Gravity countermeasure device. NASA photo 30 Jan. 1992; STS042-27-037. Courtesy of the National Aeronautics and Space Administration (NASA), Permissions: NASA Media Usage Guideline (https://www.nasa.gov/multimedia/guidelines/index.html).

Artificial gravity (AG), although popularized by science fiction, offers a compelling approach to counteract the effects of microgravity exposure on the human body and the eye. AG aims to simulate the Earth’s gravitational forces, and is proposed with a centrifuge (Fig. 1) [10] or rotating spacecraft. Previous ground human research has shown that AG allows for effective stimulation of antigravity muscles, cardiovascular system and vestibular system [11]. AG would also lessen the cephalad fluid shifts encountered in microgravity, allowing for the physiological fluid distribution to be closer to that on the ground.

The Japanese Space Agency (JAXA) recently developed a Multiple Artificial-Gravity Research System (MARS) which can simulate partial gravitational environments such as Mars and the Moon. Mao et al. [12] conducted a controlled experiment involving nine-week-old male C57BL/6 mice launched into space for a 35-day mission. These mice were subjected to two conditions aboard the International Space Station (ISS): ambient microgravity and 1 G artificial gravity generated by a centrifuge [12]. Control groups were kept on Earth under similar conditions sans the gravity variations [12]. Findings of this experiment revealed that microgravity conditions induced significant apoptosis in retinal vascular endothelial cells; approximately 64% greater than the habitat control group [12]. Proteomic analysis revealed alterations in protein expression associated with cell death, repair, inflammation, and metabolic stress, which suggests a significant impact of spaceflight on retinal structure and function [12]. Interestingly, the application of 1 G AG seemed to partially mitigate these effects [12]. Overall, this investigation found that orbital microgravity flight triggers retinal apoptosis and modifies protein expression, potentially contributing to a compromise in ocular/retinal health [12]. The mitigative effect of artificial gravity suggests a promising countermeasure to protect astronauts’ ocular health during prolonged spacecraft missions [12]. We are waiting for partial gravity animal study results to find if there is a G threshold for ocular health.

While lower body negative pressure has been explored as a countermeasure for SANS [13, 14], AG with some time schedule may potentially be a more effective countermeasure. A synergistic approach with other countermeasures can potentially mitigate SANS more effectively and enhance overall efficacy.

It is important to consider the difficulties of incorporating artificial gravity technology into spacecraft design. Spacecraft design would need to trade volume and mass from an AG system with other countermeasure systems. Reliability as a large rotating mechanism is an important factor. Securing funding for testing and deployment of AG systems in spaceflight also remains a critical challenge to advance this technology, especially when the ISS is closer to its decommissioning. The safety and efficacy of AG and the potential impact on ocular health, vestibular function, cardiovascular health and bone density should be verified before AG can be deployed including long-duration flights to Mars. Other considerations such as potential habituation effects, individual variations in responses and the long-term sustainability of AG interventions must be addressed to ensure effectiveness in the space/surface environment. Finally, robust structural and functional methods of detecting subtle spaceflight-associated ocular changes are required to fully assess the impact of AG for SANS [15, 16].
