# Correction: Artificial gravity as a potential countermeasure for Spaceflight Associated Neuro-Ocular Syndrome

**DOI:** 10.1038/s41433-024-03458-7

**Published:** 2024-11-14

**Authors:** Ethan Waisberg, Joshua Ong, Mouayad Masalkhi, Kazuhito Shimada, Andrew G. Lee

**Affiliations:** 1 Department of Clinical Neurosciences, University of Cambridge, Cambridge, UK; 2 Michigan Medicine, University of Michigan, Ann Arbor, MI USA; 3 University College Dublin School of Medicine, Belfield, Dublin Ireland; 4 TSUKUBA KOKEN, Tsukuba, Japan; 5 Department of Ophthalmology, Blanton Eye Institute, Houston Methodist Hospital, Houston, TX USA; 6 The Houston Methodist Research Institute, Houston Methodist Hospital, Houston, TX USA; 7 Departments of Ophthalmology, Neurology, and Neurosurgery, Weill Cornell Medicine, New York, NY USA; 8 Department of Ophthalmology, University of Texas Medical Branch, Galveston, TX USA; 9 University of Texas MD Anderson Cancer Center, Houston, TX USA; 10 Texas A&M College of Medicine, Bryan, TX USA; 11 Department of Ophthalmology, The University of Iowa Hospitals and Clinics, Iowa City, IA USA

**Keywords:** Eye diseases, Technology

Correction to: *Eye* (2024) 38:2847-2848

10.1038/s41433-024-03178-y; Article published online 14 June 2024

Affiliation 1 has been corrected from “Department of Ophthalmology, University of Cambridge, Cambridge, UK” to “Department of Clinical Neurosciences, University of Cambridge, Cambridge, UK”. The original article has been corrected.

